# Perceptions about tuberculosis and perceived tuberculosis-related stigma and associated factors among the mining community in Eswatini

**DOI:** 10.4314/ahs.v22i1.64

**Published:** 2022-03

**Authors:** Charles Maibvise, Mduduzi Shongwe, Vama Jele, Priscilla Dlamini, Wisdom Chiviya

**Affiliations:** 1 University of Eswatini Faculty of Health Sciences, General Nursing; University of Eswatini; 2 University of Eswatini, Midwifery Science; 3 Swaziland Migrant Mine Workers Association; 4 University of Eswatini Faculty of Health Sciences; 5 Botho University, Faculty of Health and Education

## Introduction and background

Tuberculosis (TB) remains a major global public health burden, accounting for 10 million new infections and 1.5 million deaths in 2019, making it to be among the top ten causes of death, globally, in that year^1^. The burden of TB is higher in low- and middle-income countries, especially in countries with the highest HIV and AIDS epidemic, like Eswatini1. For example, in 2017, the TB incidence rate was estimated to be 398 per 100,000 population in Eswatini^2^, way above the global average of 130 per 100,000 population1. The success of the global vision of ending TB by 2035^3^ is a function of the effectiveness of individual country efforts, especially those that form the epicentre of the TB pandemic^4^,^5^.

A successful fight against TB requires recognising and combating TB-related stigma and discrimination – one of the major social determinants of health^4^,^6^. Stigma is defined as “a complex social phenomenon based on a relationship between an attribute and a stereotype that assigns undesirable labels, qualities, and behaviours to a person with that attribute”. The roots of TB-related stigma and discrimination can in part be attributed to policies that label people living with TB as a public health hazard8. The emergence of drug-resistant TB strains, as well as the flagged relationship between TB and the already stigmatised marginalisation, low socioeconomic status, and HIV and AIDS, among other factors, have compounded the situation^9^.

Evidence shows that TB-related stigma undermines remedial efforts by compromising affected individuals and communities' health-seeking behaviours^10^. Due to fear of embarrassment secondary to anticipated TB-related prejudicial treatment in health facilities, clients end up delaying reporting TB-related clinical manifestations and/or resort to complementary and alternative medicines^8^,^9^,^11^. Quite often, key populations are primary victims of such scenarios. The mining community is one major key population when it comes to the TB pandemic, with incidences said to be three to eight times higher than in the general population^12^. The mining sector constitutes a significant proportion of Africa's gross domestic product (GDP), with the greatest amount of mining activities occurring in Southern Africa, particularly in the Republic of South Africa (RSA) where the industry employs over 500,000 people^12^,^13^.

As such, a focus on this sub-population in the fight against TB in the region is an imperative move. Studies have shown lack of knowledge, myths, and misconceptions about TB among the general population and health workers in various contexts^16^–^18^. These influence individuals' perceptions about TB and TB-related stigma^19^–^21^. Other documented factors associated with TB-related stigma include age, gender, religion, lack of social support, low level of education, and belief that TB increases the chance of getting AIDS^21^,^22^, but these have not been confirmed among the population under investigation. Thus, studies focusing on this sub-population are warranted.

The success of this move is fostered by, among other factors, a clear understanding and addressing of TB-related stigma as a critical determinant of health.

## Problem statement

The magnitude of the burden of TB-related stigma and its impact on health among the mining community in Eswatini has not been well documented. The existence of TB-related stigma as a barrier to health care delivery among the RSA mining community has been reported qualitatively but has not been quantified^14^. Similarly, in Eswatini, the problem has been studied in the general population^15^, but not among the mining community.

The level of stigma in this population is crucial in devising remedial measures.

## Purpose and objectives of the study

The purpose of this study was to describe the perceptions about TB, perceived TB-related stigma, and its associated factors among the mining community in Eswatini. The specific objectives were to: 1) describe the mining community's perceptions about TB, 2) Ddescribe perceived TB-related stigma among the mining community, and 3) Iidentify factors associated with perceived TB-related stigma among the mining community.

## Methods

Eswatini is a lower-middle-income country with a population of about 1.1 million^23^, and almost surrounded by the RSA. The country is faced with the highest HIV prevalence (27.3% among adults aged 15–49), a TB incidence rate of 398 cases per 100,000 population, as well as the second-highest prevalence of TB and HIV co-infection (at 70%) in the world^2^,^3^,^5^. The country also has severe shortage of financial and human resources for health, and very few (38%) health facilities accredited to initiate TB treatment^16^. Eswatini's mining industry is viable, contributing significantly to the country's GDP^17^,^18^. In addition to the local mining activities, an estimated 6,000 Swazi nationals work in the RSA mines. Thus, there is significant overlap and bilateral influences between the RSA and Eswatini in terms of experiences and perceptions of the mining community. Overall, there are also about 22,000 ex-mineworkers in Eswatini.^24^.

A quantitative cross-sectional descriptive study design was employed. The study was conducted as part of a broad multinational study aimed at assessing the knowledge, attitudes, and practices on TB, HIV, and Silicosis in the mining sector in Southern Africa^16^.

### Population

This study targeted current mineworkers, ex-mineworkers, and their family members, including those whose mining experiences were acquired in the RSA. To be included in the study, respondents had to be aged 18–59 years, be a member living in a selected household for at least 12 months, be present at the time of the interview, be a current mineworker or ex-mineworker, or be a family member of an ex-mineworker or current mineworker. Those who had physical/mental/emotional disabilities which precluded their ability to participate in the study were excluded.

### Sampling procedures

Multiple sampling techniques were applied, whereby, we first stratified the sample according to the four administrative regions of the country, proportionately to their population to ensure equitable representation. All mines in each region were listed, and the proportionate sample for each region was further stratified according to the mines in the region. Participants were recruited from these mines and their surrounding communities. Interval sampling was used to recruit the required number of participants from each mine using the total number of employees as the sampling frame. With help from community leaders, snowball and purposive sampling were used to locate and select eligible households which had mineworkers or ex-mine workers. The mining community members tend to know each other within an area or village, thus, participant helped to identify other eligible homesteads. In households with more than one eligible rspondent, the Kish grid method^25^ was used to select one participant.

### Sample size

The sample size was calculated using a single population proportion formula which indicated that for Eswatini, an estimated minimum of 130 participants was required. Accounting for potential non-response, a 20% contingency sample was added. Data from the five participants enrolled for pre-testing the tool were also included in the main analysis, making the total sample size to be 163.

### Instrument and data collection procedures

A well-structured researcher-administered electronic questionnaire was used. The questionnaire was programmed both in English and SiSwati into Survey-To-Go, a computer-assisted personal interviewing tablet survey software^26^. Data were collected in January and February 2017 through face-to-face interviews conducted by trained interviewers.

### Instrument validity and reliability

The questionnaire was adapted from the standard WHO Stop TB Partnership and the UNIADS questionnaire, scrutinised by experts and deemed valid and reliable for multinational use16. It was further pretested in all participating countries and confirmed to be valid and reliable. Likewise, pretesting for local use in Eswatini was also conducted among five participants, and no flaws were identified. Research assistants were trained to standardise the process of data collection. Data were captured electronically onto a central server to minimise transfer errors.

### Dependent variable

Perceived TB-related stigma was assessed using seven carefully selected questions from the main questionnaire (see Table 3). Each question was rated such that a score of one upwards (depending on the number of response options per question) was assigned to stigmatizing responses and a score of zero was assigned to non-stigmatizing responses. Thereafter, the responseswere summed up to generate a TB-related stigma score ranging from 0 to 15. The composite score was then dichotomized using its median (which was 5.0) as a cut-off value with scores above the median value coded as 1 indicating high TB-related stigma, and scores below or equal to the median value coded as 0 indicating low TB-related stigma.

**Table 3:** Perceived TB-related stigma among mining communities in Eswatini (*N*=163)

Variable	*n*	%
**Think TB would affect your social relations**		
Yes	98	60.1
No	53	32.5
Don't know	12	7.4
**Think TB would affect your work relations**		
Yes	118	72.4
No	45	27.6
**If on TB treatment would not like people to know**		
Yes	78	47.9
No	82	50.3
Don't know	3	1.8
**Can access TB services without fear of being discriminated**		
Yes	143	87.7
No	16	9.8
Don't know	4	2.5
**My family would support me if on TB treatment**		
Yes	159	97.5
No	4	2.5
**Feelings about people with TB**		
“I feel compassion and desire to help.”	152	93.3
“I feel compassion but I tend to stay away from these people.”	3	1.8
“I fear them because they may infect me.”	7	4.3
“I have no particular feeling.”	1	0.6
**In your community, how is a person who has TB usually regarded/treated?**		
Most people reject him or her	8	4.9
Most people are friendly, but generally try to avoid him/her	25	15.3
The community mostly supports and helps him or her	97	59.5
The government or national programs would take care of them	7	4.3
Other	26	16

### Explanatory variables

Explanatory variables were selected from sociodemographic variables in the questionnaire, based on the literature review^10^,^20^–^22^. These included age, sex, region of residence, length of stay in the community, religion, current marital status, educational level, and perceptions about TB, amongst others.

### Data analysis

All statistical procedures were performed using the Statistical Package for Social Sciences (SPSS) version 25.027. Descriptive analyses included computing frequencies and proportions for categorical data, whereas, bivariate (simple) binary logistic and multiple binary logistic regression models were fitted using the augmented backward elimination strategy^28^ to determine factors associated with high or low TB-related stigma. Crude odds ratios (CORs), adjusted odds ratios (AORs), and their 95% confidence intervals (CIs) were computed as measures of association. All significance tests were two-sided and at an alpha of .05.

### Validity and reliability

#### Ethical considerations

Ethical clearance was granted by the Eswatini National Health and Human Research Review Board. Written permissions to conduct the study were sought from each Regional Administrator and from the Commissioner of Mines. Permissions were also sought from the authorities of participating mines as well as traditional leaders of each participating community. Further, the four basic principles of research ethics were enforced as follows:

#### Respect of persons and human dignity

Informed consent was obtained from each participant before data collection. Participants were made aware that their participation was voluntary and they were free to withdraw from the study at any time or refuse to answer any question without any penalty. No names were collected on the questionnaire for anonymity. A quiet and private room or space was requested at each site of data collection to ensure privacy and confidentiality. After each interview, the data were instantly uploaded onto the central server, accessible only to the statistician.

#### Justice

Rigorous probability sampling techniques were applied to ensure a fair selection of participants. Participants who were incapable of effective participation owing to any debilitating physical, mental, or emotional ailment were excluded from the study. Data collectors honoured their promises and obligations to participants, including timekeeping as well as maintaining anonymity, privacy, and confidentiality. Researchers' contact details were availed to participants for possible subsequent follow-up queries.

#### Beneficence

Participants were made aware that there were no direct individual benefits associated with participating in this study. Instead, recommendations based on the study would help improve the health and wellbeing of the mining community at large, themselves included, particularly on issues relating to TB.

#### Nonmaleficence

this study involved no invasive procedures and hence posed no biomedical risk to participants. Questions were also scrutinised to ensure that none of them were psychological or emotionally traumatic. In any case, measures were put in place to ensure instant referrals to counsellors and psychotherapists in the event of such unforeseeable adverse effects.

## Results

Table 1 below shows selected demographic characteristics of the 163 participants who were enrolled.

**Table 1:** Socio-demographic characteristics of participants (*N*=163)

Variable	*n*	%
**Age in years**		
19–35	52	31.9
36–59	111	68.1
**Sex**		
Female	50	30.7
Male	113	69.3
**Residence**		
Rural	138	84.7
Urban	10	6.1
Peri-urban	15	9.2
**Current marital status**		
Married	101	62.0
Not married	62	38.0
**Highest education level completed**		
No school	12	7.4
Elementary/primary	46	28.2
Secondary/High school	92	56.4
Tertiary	13	8.0
**Length of stay in current community**		
< 6 years	23	14.1
≥ 6 years	140	85.9
**Mining community status**		
Currently a mineworker	62	38.0
Ex-mineworker	42	25.8
Family member of a current/ex-mineworker	59	36.2
**Mined commodity**		
Coal	42	25.8
Gold	66	40.5
Other	55	33.7
**Reason for leaving employment** (*n*=91)		
Medically boarded*	38	41.8
Fired/company closed/voluntary left/retired	36	39.6
Other	17	18.7

* 50% (*n*=19) of these were boarded due to TB

### Perceptions about Tuberculosis

Majority of the participants knew people who had TB and they considered TB to be a serious disease in the mines. Table 2 shows a compilation of participants' responses to questions on perceptions about TB.

**Table 2:** Perceptions of the mining community about tuberculosis (*N*=163)

Variable	n	%
**How serious of a disease is TB?**		
Very serious	138	84.7
Somewhat serious	18	11.0
Not very serious	7	4.3
**How serious of a disease is TB in the mines?**		
Very serious	147	90.2
Somewhat serious	13	8.0
Not very serious	3	1.8
**Do you think you can get TB?**		
Yes	159	97.5
No	4	2.5
**Reaction if you would find out you had TB**		
Accept it	9	5.5
Fear	31	19.0
Surprise	29	17.8
Shame	3	1.8
Embarrassed	0	0.0
Sadness or hopelessness	18	11.0
Don't know	73	44.8
**Know people who have/had TB**		
Yes	144	88.3
No	19	11.7
**Think that a person with HIV should be concerned about TB**		
Yes	151	92.6
No	11	6.7
Don't know	1	0.6
**What worries you most when you think about TB?**		
Likelihood of being HIV positive	15	9.2
Likelihood of contracting the disease	74	45.4
Not being able to get treatment	27	16.6
TB may decrease my chance of employment	4	2.5
Other	43	26.4
**Who you would talk to about your illness if you had TB**		
A health professional	42	25.8
Spouse	65	39.9
Parent	25	15.3
Other	30	18.4
None	1	0.6

Table 3 summarises participants' responses to selected questions depicting TB related stigma.

Overall prevalence of perceived TB-related stigma: As shown in Figure 1, 27.6% (n=45) of the participants were classified as having high perceived TB-related stigma.

Factors associated with perceived TB-related stigma: In bivariate analyses, participants who had stayed for six years or longer in a community had higher odds of having high TB-related stigma than those who stayed less than six years, COR=4.66 (95% CI: 1.05, 20.74). In multiple logistic regression analysis, those aged 19–35 years (AOR=1.65, 95% CI: 1.01, 5.44) vs those aged 36–59 years, and those who had stayed six years or longer in a community (AOR=6.38, 95% CI: 1.36, 29.8) compared to those who had stayed less than six years, had higher odds of having high TB-related stigma, adjusting for the other variables in the model. On the contrary, participants from the Manzini region had lower odds of having high TB-related stigma compared to those in the Lubombo region, adjusting for the other variables in the model, AOR=0.20 (95% CI: 0.05, 0.89). Table 4 illustrate these findings.

## Discussion

Nearly a third (27.6%) of the participants had high perceived TB-related stigma, similar to findings by Courtwright and Turner^29^ who found that the prevalence of TB-related stigma among at-risk populations in different communities ranged from 27% to 80%. They^29^ opined that such high levels of TB-related stigma could be attributed in part to having a positive pulmonary TB diagnosis^30^. Even though we did not elicit the participants' past or current TB status in our study, the fact that only 34% of participants had ever sought TB services suggests that the majority had never been diagnosed with TB, which could explain the relatively low prevalence of high TB-related stigma in our sample.

.....found that those aged 35 years and below had higher odds of having high TB-related stigma. Even though this association is not well documented in the literature, one study found no association between the two variables, but in that study, the population was homogenously young (10 to 14 years)^31^. Duko et al.^30^, in a study of participants with a mean age of 32.3 years, found a TB-related stigma prevalence of 42.4%. In the current study, the mean age was 44 years, which could explain the relatively low level of perceived TB-related stigma.

This study also revealed that TB is generally perceived as a serious disease among the studied community, consistent with a study by Mbuthia and Olungah^32^. However, this perception did not significantly correlate with TB-related stigma. Participants indicated that it was neither a shame nor an embarrassment to have TB, implying that a large sector of the mining community may not necessarily be stigmatising themselves since shame and embarrassment are said to be key indicators or manifestations of internal stigma^33^. This finding is inconsistent with what has been observed among people living with HIV, TB, and epilepsy, whereby, these subgroups have been found to stigmatize themselves more than other people stigmatise them^34^. Perhaps in our sample, this was because the majority (95.7%) were aware that TB can be cured, and that treatment was readily available at their local clinics.

When asked how they would react if they were to be diagnosed with TB, participants reported fear as one of the initial reactions, similar to findings by Mukerji and Turan^35^ who found that fear of loss of friends and family were some of the clinical manifestations of TB-related stigma. However, in our case, we did not solicit the source of fear, and we implore future studies to investigate further. Furthermore, slightly above a third of the participants in this study reported having ever accessed TB services. This proportion is relatively low, considering that more than 70% of them said they lived within a 10 km radius from the nearest health facility. Besides, it is expected and encouraged that at-risk populations undergo periodic TB screening^36^. The apparent poor health-seeking behaviour coupled with the observed traces of fear in this study potentially signify the prevalence of some levels of stigma among the mining community. TB-related stigma may inhibit health-seeking behaviour through fears of negative treatment, fear of being disparaged, gossiped or isolated by the community, and fear of negative stereotypes^34^.

Fear of the community and the risk of being disparaged may in part also explain the statistically significant association observed between duration of stay in a community and perceived TB-related stigma, similar to findings from a study exploring correlates of perceived stigma towards leprosy in Thailand^22^. The longer one stays in a community, the more people know him/her, and inferentially, this may increase the likelihood of being gossiped about. This could also reflect the persistence of stereotypical views towards TB among people who have stayed longer in a community, or it could also reflect a lack of stigma reduction strategies such as health education in the studied community^22^.

The proportions of people who thought their communities would reject or avoid them or who were confident that the community would support them if they could be infected with TB is a cause for concern, given that a community is expected to provide a compassionate environment free of stigmatization and discrimination^4^.

This study also revealed a statistically significant association between geographical location and perceived TB-related stigma, consistent with an earlier systematic review^29^ which found that that stigma was more prevalent in rural than urban areas. In our study, the mining communities interviewed were located in both rural and urban areas. It remains unclear why participants in the Manzini region had lower TB-related stigma scores, but it could be that this region has a fair mix of both urban and rural areas. More studies are needed to investigate this finding further.

A notable proportion of the participants reported that they thought being diagnosed with TB would affect their social and work relations. This is perhaps due to that most policies and/or health education about TB portray people living with TB as public health hazards who must be avoided8. Cognizant of this stance, the WHO recommended that individuals' TB status should be kept privatand confidential as a way of combating stigma^4^. On the other hand, such secrecy may also portray an impression of undesirability, and thus perpetuate stigma. Further, in the general context of communicable diseases, disclosure is the gateway to any remedial measures by any potential contributor in any setting, as opposed to secrecy, privacy, and confidentiality^37^,^38^.

## Strengths and limitations

This study has some strengths and limitations. First, to our knowledge, this is the first study to investigate TB-related stigma among the mining communities in Eswatini. Second, the study used multistage probability sampling methods which enhanced its external validity. Third, we used well-validated items which minimised measurement bias. However, the cross-sectional nature of the study limits its ability to establish causality between the studied explanatory variables and the dependent variable. The relatively small sample size contributed to the wide confidence intervals in the regression models, thus studies with more statistical power are warranted. Lastly, social desirability bias cannot be completely ruled out due to the nature of the social variables asked in this study.

## Conclusion and recommendations

In this study, we found that the mining community perceives TB as a serious disease and that they also appreciated that, like any other disease, those infected with, and/or affected by, Tb still deserve to be treated like any other person, without prejudice. However, we also found that nearly a third of the respondents had high levels of perceived TB-related stigma, especially younger participants and those who had lived longer in their communities. Therefore, efforts to combat TB-related stigma among the mining community should target younger members and those who have stayed longer in their communities to alleviate any stereotypic behaviours and thoughts among mining communities. Health education, accurate messaging, and demystification of misconceptions about TB remain key in combating TB-related stigma. Mining communities should also be encouraged to engage in “End TB” activities in a compassionate and non-stigmatising manner to earn trust from each other. Future studies should explore the reasons behind the internal stigma, potential external stigma, and their sources among mining communities, as well as the reasons for the geographic and age disparities with regards to TB-related stigma among members of this community. Lastly, future studies with large sample sizes may consider conducting factor or cluster analysis to determine which perceptions affect stigma within mining communities.
